# Herpes Simplex Virus 1 Infection Promotes the Growth of a Subpopulation of Tumor Cells in Three-Dimensional Uveal Melanoma Cultures

**DOI:** 10.1128/JVI.00700-18

**Published:** 2018-09-12

**Authors:** Tibor Valyi-Nagy, Brian Fredericks, Aditya Ravindra, James Hopkins, Deepak Shukla, Klara Valyi-Nagy

**Affiliations:** a Department of Pathology, University of Illinois at Chicago, College of Medicine, Chicago, Illinois, USA; b Department of Ophthalmology and Visual Sciences, University of Illinois at Chicago, College of Medicine, Chicago, Illinois, USA; c Department of Microbiology and Immunology, University of Illinois at Chicago, College of Medicine, Chicago, Illinois, USA; Northwestern University

**Keywords:** herpes simplex virus, oncolytic virotherapy, three-dimensional culture, melanoma, growth promotion

## Abstract

Herpes simplex virus 1 (HSV-1)-mediated oncolytic therapy is an emerging cancer treatment modality with potential effectiveness against a variety of malignancies. To better understand the interaction of HSV-1 with neoplastic cells, we inoculated three-dimensional (3D) cultures of human uveal melanoma cells with HSV-1. 3D melanoma cultures were established by placing tumor cells on the surface of a Matrigel matrix, which was followed by the growth of tumor cells on the matrix surface and invasion of the Matrigel matrix by some tumor cells to form multicellular tumor spheroids within the matrix. When established 3D melanoma cultures were inoculated with HSV-1 by placing virus on the surface of cultures, virus infection caused extensive death of melanoma cells growing on the surface of the 3D matrix and significantly decreased the number of tumor cell spheroids within the matrix. However, HSV-1 infection did not lead to a complete destruction of tumor cells in the 3D cultures during a 17-day observation period and, surprisingly, HSV-1 infection promoted the growth of some melanoma cells within the matrix as determined by the significantly increased size of residual viable multicellular tumor spheroids in virus-inoculated 3D cultures at 17 days after virus inoculation. Acyclovir treatment inhibited HSV-1-induced tumor cell killing but did not block the virus infection-induced increase in spheroid size. These findings suggest that although HSV-1 oncolytic virotherapy may cause extensive tumor cell killing, it may also be associated with the unintended promotion of the growth of some tumor cells.

**IMPORTANCE** Cancer cells are exposed to HSV-1 during oncolytic virotherapy with the intention of killing tumor cells. Our observations reported here suggest that potential dangers of HSV-1 oncolytic therapy include promotion of growth of some tumor cells. Furthermore, our findings raise the possibility that HSV-1 infection of neoplastic cells during natural infections or vaccinations may promote the growth of tumors. Our study indicates that HSV-1 infection of 3D tumor cell cultures provides an experimental platform in which mechanisms of HSV-1-mediated promotion of tumor cell growth can be effectively studied.

## Abstract (precis)

Cancer cells are exposed to HSV-1 during oncolytic virotherapy with the intention of killing tumor cells. Our observations reported here suggest that potential dangers of HSV-1 oncolytic therapy include promotion of growth of some tumor cells. Furthermore, our findings raise the possibility that HSV-1 infection of neoplastic cells during natural infections or vaccinations may promote the growth of tumors. Our study indicates that HSV-1 infection of 3D tumor cell cultures provides an experimental platform in which mechanisms of HSV-1-mediated promotion of tumor cell growth can be effectively studied.

## INTRODUCTION

Oncolytic virotherapy is an emerging cancer treatment modality with potential effectiveness against a variety of malignancies (1). Various strains of herpes simplex virus 1 (HSV-1) serve as prominent examples of promising oncolytic agents. For example, talimogene laherparepvec (T-VEC), an FDA-approved modified HSV-1 oncolytic virus, has been shown to suppress the growth of advanced malignant melanoma in humans, and G47delta, a third-generation oncolytic herpesvirus, exhibited efficiency in numerous *in vivo* solid tumor models, including glioma, breast, and prostate cancers (1, 2). Oncolytic HSV-1 therapy is dependent upon virus replication in tumor cells and is augmented by host antiviral and infection-induced antitumor immune responses (1, 3–6). In T-VEC, deletions of wild-type γ*34.5* and α*47* viral genes promote targeting tumor cells over nonneoplastic cells and enhance the body's natural antitumor response (7).

In spite of significant progress, oncolytic virotherapy, including HSV-1-mediated oncolytic therapy, faces significant challenges. Factors that may limit the effectiveness of HSV-1 oncolytic therapy include restricted intratumoral spread of oncolytic virus, activation of intracellular tumor defenses that limit virus-induced tumor cell killing, and quick virus clearance by the host immune system (1, 8–12). Potential dangers of HSV-1 oncolytic therapy include virus-mediated damage to nonneoplastic tissue and promotion rather than inhibition of tumor growth. Several aspects of HSV-1-tumor cell interactions have been difficult to study *in vitro*, as traditional two-dimensional (2D) tumor cell cultures are typically very sensitive to virus-mediated destruction and are quickly destroyed, unlike in the *in vivo* situation, where tumor cell destruction by HSV-1 is often incomplete (12, 13).

Three-dimensional (3D) tumor cell cultures provide a useful *in vitro* experimental platform to study many aspects of tumor growth and cancer therapy (14–22). Compared to traditional two-dimensional monolayer culture studies, 3D cultures have been shown to better simulate *in vivo* cellular behaviors such as growth, differentiation, invasion, and apoptosis (14–20). 3D cell cultures have also proven to be an effective design to study the interaction of HSV with tumor cells (12, 13, 23, 24). Melanoma cells grown in 3D are more resistant to HSV-1 infection than cells grown in 2D, and HSV-1 may establish a quiescent infection in some melanoma cells in 3D cultures (12, 13).

To further investigate the mechanism by which HSV-1 interacts with neoplastic cells, in the current study we inoculated 3D OCM-1 human uveal melanoma cultures with an HSV-1 strain, K26GFP, which expresses the green fluorescent protein (GFP) when it replicates (25). Recombinant HSV-1 K26GFP was derived from wild-type HSV-1 strain KOS and grows as a wild-type virus in cell culture (25). We found that although HSV-1 infection caused extensive tumor cell killing in the 3D cultures, it also promoted the growth of a subpopulation of invasive tumor cells, suggesting that HSV-1 oncolytic virotherapy can potentially promote tumor growth *in vivo*.

## RESULTS

### Uveal melanoma cells placed on the surface of 3D Matrigel matrix grow on the Matrigel surface and invade the matrix to form multicellular spherical tumor cell aggregates—spheroids.

**FIG 1:**
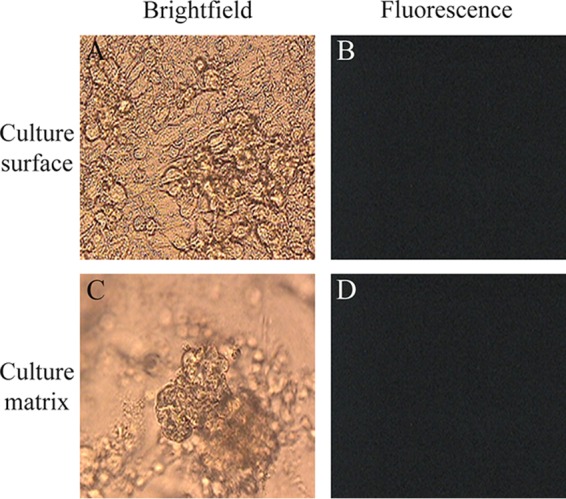
Morphology of cultures and lack of HSV-1 infection-mediated GFP expression in OCM-1 3D uveal melanoma cultures 17 days after mock infection. (A) Brightfield image of 3D culture surface; (B) fluorescence image of 3D culture surface with no evidence of GFP expression; (C) brightfield image of 3D culture matrix with multicellular spheroids; (D) fluorescence image of 3D culture matrix with no evidence of GFP expression (magnification, ×400).

After OCM1 uveal melanoma cells were placed on the surface of Matrigel matrix, the cells grew on the Matrigel surface and began invading the matrix, where they formed spherical tumor cell aggregates, termed spheroids. As the cultures were observed daily, the spheroids appeared to be growing in size and cells on the surface of Matrigel became increasingly crowded, but cultures could be maintained for several weeks. 3D melanoma cultures exposed to mock infection grew similarly to uninfected 3D cultures, and no GFP expression (fluorescence) was observed either on the surface or within the matrix of the mock-infected 3D cultures (Fig. 1 and Table 1). This observed pattern of OCM1 uveal melanoma growth in 3D cultures was similar to previous published observations (12).

**TABLE 1:** Following placement of HSV-1 on 3D melanoma cultures, virus infects tumor cells on the culture surface and spreads to tumor cells forming spheroids in culture matrix^*a*^

Days after treatment	% of OCM-1 uveal melanoma cells expressing GFP following:			
	HSV-1 K26GFP infection alone		HSV-1 K26GFP infection + acyclovir treatment	
	Surface	Matrix	Surface	Matrix
1	5	0	0	0
2	85	0	2	0
3	80	2	2	0
4	50	5	5	0
7	5	0	5	0
8	0	0	5	0
9	0	0	5	0
10	0	5	5	0
11	0	10	0	0
13	0	20	0	5
14	0	5	0	2
15	0	5	0	10
16	0	15	0	20
17	0	5	0	10

a The observation period was 17 days. Mock-infected cultures did not show GFP expression at any time.

### Following placement of HSV-1 on 3D melanoma cultures, virus infects tumor cells on the culture surface and spreads to tumor cells forming spheroids in culture matrix.

**FIG 2:**
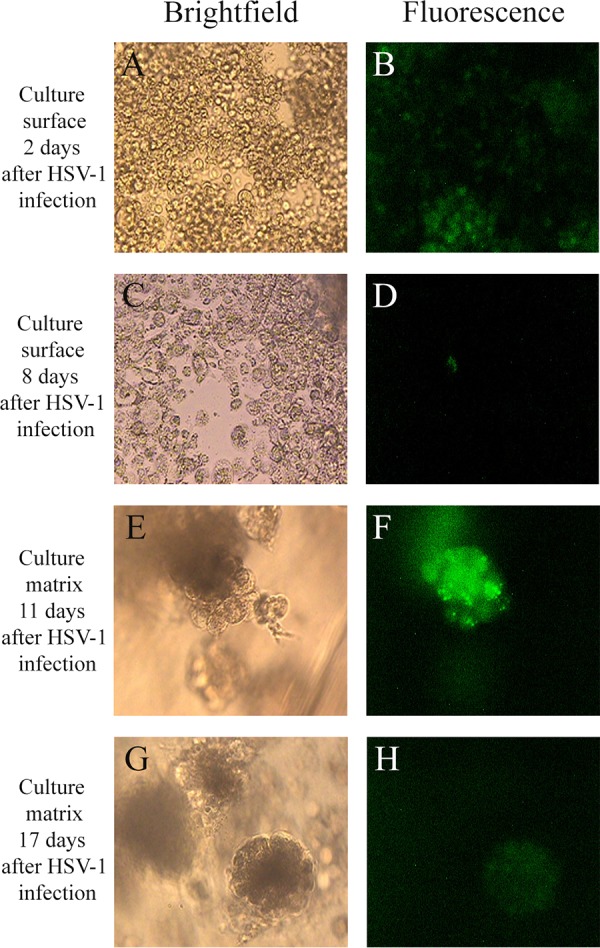
Morphology of cultures (A, C, E, G) and HSV-1 infection-mediated GFP expression (B, D, F, H) in OCM-1 3D uveal melanoma cultures following HSV-1 K26GFP infection. (A and B) Brightfield (A) and fluorescence (B) images of 3D culture surface 2 days after HSV-1 K26GFP infection; (C and D) brightfield (C) and fluorescence (D) images of 3D culture surface 8 days after HSV-1 K26GFP infection (magnification, ×200 for panels A through D); (E and F) brightfield (E) and fluorescence (F) images of 3D matrix 11 days after HSV-1 K26GFP infection; (G and H) brightfield (G) and fluorescence (H) images of 3D matrix 17 days after HSV-1 K26GFP infection (magnification, ×400 for panels E through H).

**FIG 3:**
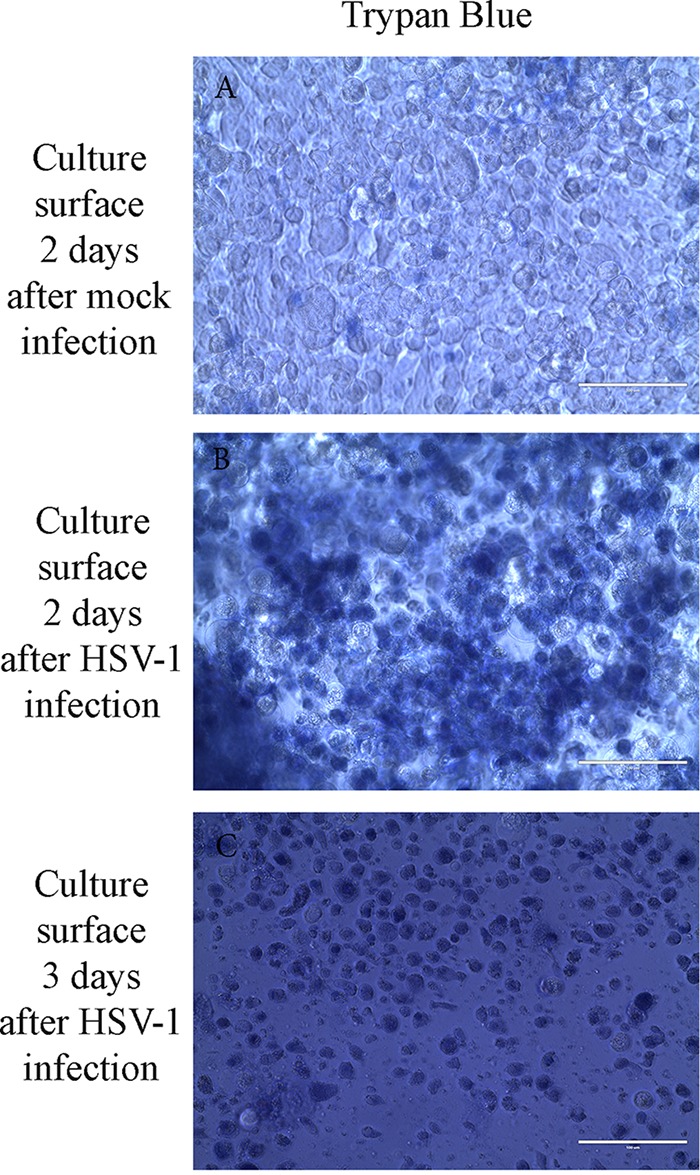
Cell death on the surface of 3D uveal melanoma cultures following HSV-1 infection. (A) No significant uptake of Trypan blue by cells on the surface of 3D uveal melanoma cultures 2 days after mock infection. (B) Many cells demonstrate uptake of Trypan blue consistent with cell death on the surface of 3D uveal melanoma cultures 2 days after HSV-1 K26GFP infection. (C) Extensive uptake of Trypan blue by cells on the surface of 3D uveal melanoma cultures 3 days after HSV-1 K26GFP infection. Bars, 100 μm.

When 3D melanoma cultures were inoculated with HSV-1 by placing virus on the surface of the cultures, infection first caused significant expression of GFP in cells present on the culture surface (Fig. 2B) and led to the death of many of these cells as documented by the uptake of Trypan blue (Fig. 3B and C). At 2 days after virus inoculation, 80% of melanoma cells on the culture surface expressed GFP consistent with extensive virus replication. At day 3, most cells on the surface appeared to be destroyed (Fig. 3C). Surface GFP expression was visible until day 8 (Fig. 2; Table 1). Virus spread to cells growing in the culture matrix with a slight delay as virus-directed GFP expression was first detected in some tumor cells forming spheroids only by 3 days following virus inoculation (Table 1). Cells in the matrix did not show significant increases in GFP expression until around day 11, when 10% of cells expressed GFP. The most extensive GFP expression was found on days 11 and 13, and it was still visible until day 17 (Table 1; Fig. 2F and H). Tumor spheroids containing viable tumor cells remained present throughout the 17-day observation period following virus inoculation.

These findings indicate that following placement of HSV-1 on 3D melanoma cultures, the virus infects and kills many tumor cells on the culture surface and spreads to tumor cells forming multicellular spheroids in the culture matrix, leading to the destruction of some tumor cells.

**FIG 4:**
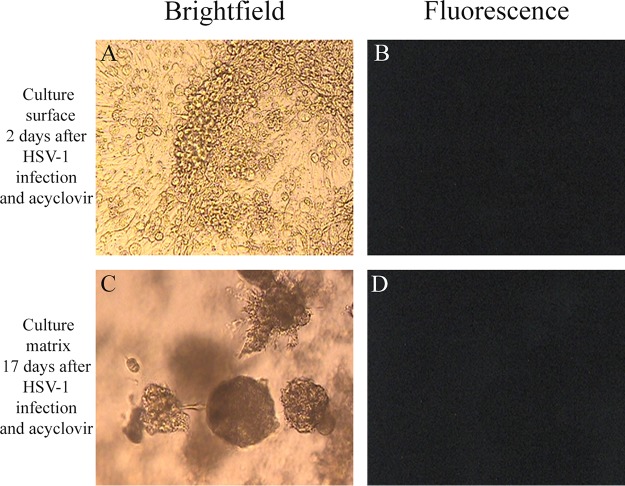
Acyclovir inhibits HSV-1 replication and spread in 3D melanoma cultures. Morphology of culture (A, C) and lack of HSV-1 infection-mediated GFP expression (B, D) in OCM-1 3D uveal melanoma cultures following HSV-1 K26GFP infection and acyclovir treatment. (A) Brightfield image of 3D culture surface 2 days after HSV-1 K26GFP infection and acyclovir treatment; (B) fluorescence image of 3D culture surface 2 days after HSV-1 K26GFP infection and acyclovir treatment with no evidence of GFP expression; (C) brightfield image of 3D matrix 17 days after HSV-1 K26GFP infection and acyclovir treatment; (D) fluorescence image of 3D culture matrix 17 days after HSV-1 K26GFP infection and acyclovir treatment with no evidence of GFP expression (magnification, ×200).

In cultures that were infected with HSV-1 K26GFP and were also treated with acyclovir, spread of HSV-1 within the 3D cultures was reduced and delayed (Table 1; Fig. 4) consistent with acyclovir inhibition of HSV-1 replication. Specifically, GFP expression among cells on the culture surface remained in the 2 to 5% range until day 9, when significant cell death was also observed. Tumor cells within the culture matrix in acyclovir-treated cultures did not show significant fluorescence until day 15 and peaked at day 16 with an observation of 20% fluorescence followed by a decline to 10% at day 17 (Table 1).

These findings indicate that acyclovir treatment inhibited and delayed the progression of HSV-1 K26GFP in 3D melanoma cultures.

### HSV-1 inoculation leads to decreased tumor spheroid number but increased average tumor spheroid size in 3D melanoma cultures.

**FIG 5:**
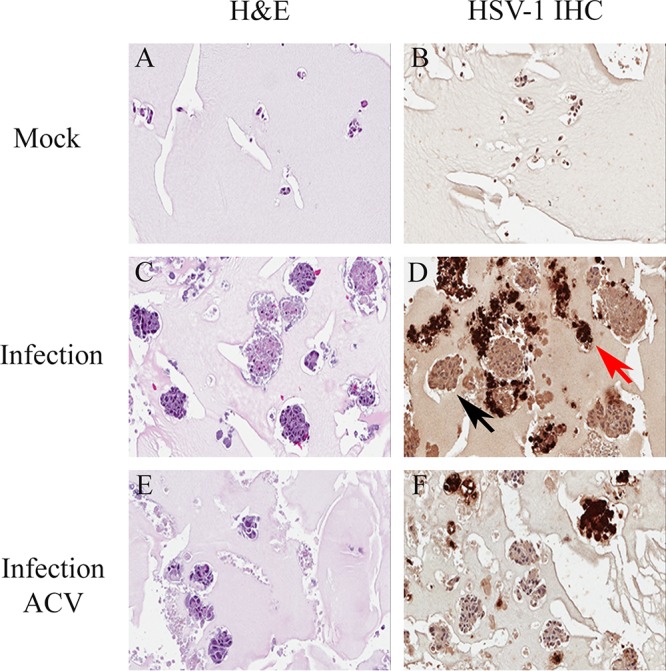
Morphology and immunohistochemical (IHC) detection of HSV-1 protein expression in 3D uveal melanoma cultures 17 days after HSV-1 or mock infection. Dark brown color in immunostained sections in panels D and F represents HSV-1 protein expression. Mock-infected control culture stained with hematoxylin and eosin (H&E) (A) or stained with HSV-1 antibody by immunohistochemistry (HSV IHC) (B). HSV-1 infected culture stained with hematoxylin and eosin (C) or stained with HSV-1 antibody (D). The red arrow in panel D points to a multicellular spheroid containing many cells expressing HSV-1 proteins. The black arrow in panel D points to a multicellular spheroid without cells expressing HSV-1 proteins. HSV-1-infected and acyclovir-treated culture stained with hematoxylin and eosin (E) or stained with HSV-1 antibody (F). Magnification, ×164.

To define the effect of HSV-1 infection on melanoma cell growth in 3D tumor cultures, 3D cultures were extracted from culture dishes, fixed, and stained with hematoxylin and eosin 17 days following virus inoculation or mock infection and were analyzed by morphometry for average tumor spheroid number and tumor spheroid size (Table 2; Fig. 5A, C, and E). The spheroids in the mock-infected cultures were significantly more numerous and were significantly smaller than spheroids in HSV-1-inoculated cultures (Table 2). Specifically, in mock-infected 3D cultures, the average spheroid number was 1.8806 ± 0.150 per 0.25-mm^2^ culture area, and the average spheroid area was 2,061.611 ± 309.928 square micrometers (Table 2). In contrast, in HSV-1-inoculated cultures, the average spheroid number was 0.503546 ± 0.080 per 0.25-mm^2^ culture area and the average spheroid was 7,036.967 ± 473.617 square micrometers (Table 2). Both of these measurements were significantly different from those for mock-infected cultures (*P* < 0.05). Relative to mock-infected cultures, spheroid numbers were not reduced in cultures inoculated with HSV-1 and also treated with acyclovir, but spheroid sizes were significantly increased (*P* < 0.05). Specifically, in HSV-1-inoculated and acyclovir-treated cultures, the average spheroid number was 1.8969 ± 0.182 per 0.25-mm^2^ culture area and the average spheroid size was 5,033.754 ± 418.453 square micrometers (Table 2). These observations indicate that HSV-1 inoculation leads to decreased tumor spheroid number but increased average tumor spheroid size in 3D melanoma cultures.

**TABLE 2:** HSV-1 inoculation leads to decreased tumor spheroid number but increased average tumor spheroid size in 3D uveal melanoma cultures^*a*^

Treatment	Avg no. of spheroids (per 0.25-mm^2^ culture area) ± SD	Avg spheroid size (mm^2^) ± SD
Mock infection	1.8806 ± 0.150	2,061.611 ± 309.928
HSV-1 K26GFP infection	0.503546 ± 0.080*	7,036.967 ± 473.617*
HSV-1 K26GFP infection + acyclovir	1.8969 ± 0.182	5,033.754 ± 418.453*

a Data were collected 17 days after infection or mock infection. *, significantly different (*P* value < 0.05) from mock infection.

These observations also indicate that inhibition of HSV-1 replication and spread in 3D melanoma cultures with acyclovir inhibits the virus-mediated destruction of tumor spheroids but does not inhibit the HSV-1-induced enhancement of spheroid size.

To determine whether the detected increase in spheroid size following HSV-1 infection was due to increased tumor cell number, we determined the average number of nuclei (nuclear profiles) per spheroid in hematoxylin and eosin (H&E)-stained sections of HSV-1-infected, HSV-1-infected and acyclovir-treated, and mock-infected 3D cultures 17 days following HSV-1 or mock infection. As tumor cells were mononuclear, nucleus counts were representative of the number of tumor cells per spheroid. These measurements indicated that the average number of nuclei and thus the average number of cells were the greatest in spheroids in HSV-1-infected cultures (17.58 ± 12.72 nuclear profiles/spheroid section [number of spheroids analyzed = 99]) and were the smallest in spheroids in mock-infected 3D cultures (4.19 ± 3.03 nuclear profiles/spheroid section [number of spheroids analyzed = 104]). The average number of nuclei in HSV-1-infected and acyclovir-treated 3D cultures was 13.37 ± 10.65 nuclear profiles/spheroid section (number of spheroids analyzed = 78). The detected differences in average nucleus number per spheroid among the different treatment groups were statistically significant (*P* < 0.05). These findings indicate that the increase in spheroid size following HSV-1 infection in 3D melanoma cultures is associated with an increased number of tumor cells in spheroids.

### HSV-1 protein expression is detected in some but not all enlarged melanoma spheroids in HSV-1-infected 3D melanoma cultures.

To better understand the role of expression of HSV-1 proteins in HSV-1-induced enhancement of melanoma spheroid size, 5-μm sections of paraformaldehyde-fixed paraffin-embedded 3D cultures collected 17 days after virus inoculation or mock infection were processed for immunohistochemistry for HSV-1 protein expression using a polyclonal anti-HSV-1 protein antibody. HSV-1 protein expression was visualized as brown staining under a light microscope and photographed (Fig. 5B, D, and F). In HSV-1-inoculated cultures, some spheroids demonstrated HSV-1 protein expression (brown staining) while many spheroids were negative for HSV-1 protein expression (Fig. 5D). HSV-1 protein expression in spheroids with positive staining ranged from focal to complete involvement of all cells forming the given spheroid (Fig. 5D). In HSV-1-inoculated and acyclovir-treated 3D cultures, some spheroids also demonstrated HSV-1 protein expression (brown staining) while many spheroids were negative for HSV-1 protein expression (Fig. 5F). In mock-infected (control) 3D cultures, spheroids were negative for brown staining, that is, for HSV-1 protein expression (Fig. 5B).

To assess the relationship of HSV-1 protein expression and spheroid size, sections were photographed using Image Scope and were analyzed in Image J to determine the intensity of immunostaining for HSV-1 proteins for whole individual spheroids as well as their size (area) by morphometry. Immunostaining intensity for spheroid profiles was determined for whole spheroid profiles and not for individual cells making up spheroids. Quantitative data for HSV-1 protein expression intensity and spheroid size were collected for at least 100 randomly selected spheroids in sections of HSV-1-infected, HSV-1-infected and acyclovir-treated, and mock-infected (control) 3D melanoma cultures collected 17 days after virus inoculation.

**FIG 6:**
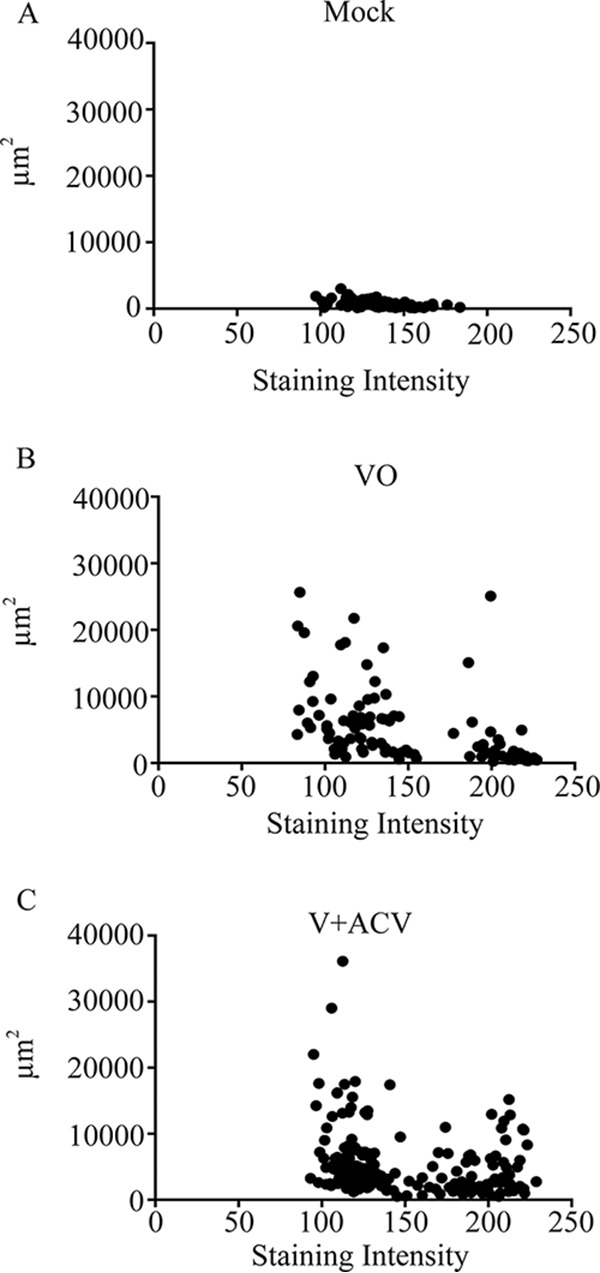
Strength of HSV-1 protein immunostaining as a function of spheroid size (in square micrometers) in mock-infected (A), HSV-1-infected (virus only [VO]) (B), and HSV-1-infected and acyclovir-treated (V + ACV) (C) 3D uveal melanoma cultures 17 days after mock or virus infection. Five-micrometer sections of 3D melanoma cultures were reacted with a polyclonal anti-HSV-1 antibody, and immunostained sections were photographed using Image Scope and analyzed in Image J to determine the strength of immunostaining. Immunostaining intensity for spheroid profiles was determined for whole randomly selected spheroid profiles and not for individual cells making up spheroids. Strength of HSV-1 protein immunostaining above the highest value of 183.66 detected in mock controls is consistent with HSV-1 protein expression. Spheroid size (area) was expressed in square micrometers. Enlarged spheroids (larger than the largest, 3,014.81-square-micrometer spheroid in mock controls) in HSV-1-inoculated and HSV-1-inoculated and acyclovir-treated cultures included both HSV-1 protein-expressing spheroids and spheroids negative for HSV-1 protein expression.

In mock-infected (control) 3D cultures, HSV-1 protein staining intensity in 103 examined whole spheroids ranged from 97.22 to 183.66 (average intensity, 138.71) and spheroid sizes ranged between 86.48 and 3,014.81 square micrometers (average spheroid size, 605.14 μm^2^) (Fig. 6A). In HSV-1-inoculated cultures, HSV-1 protein staining intensity in 102 examined whole spheroids ranged from 83.35 to 227.33 (average intensity, 149.37) and spheroid sizes ranged between 239.30 and 25,664.12 square micrometers with an average size of 5,404.7 square micrometers (Fig. 6B). Spheroid size for this population of spheroids in HSV-1-inoculated cultures was significantly increased relative to spheroids in mock-infected cultures (*P* < 0.05). Enlarged spheroids in HSV-1-inoculated cultures (larger than the largest, 3,014.81-square-micrometer spheroid in mock controls) included both HSV-1 protein-expressing spheroids (immunostaining intensity above the highest value of 183.66 detected in mock controls) and spheroids with no significant HSV-1 protein expression as demonstrated by immunostaining intensity in the range of uninfected controls (Fig. 6B). Specifically, 6 of the 55 enlarged spheroids (10.9%) expressed HSV-1 proteins.

In HSV-1-inoculated and acyclovir-treated cultures, HSV-1 protein staining intensity in 198 examined whole spheroids ranged from 93.21 to 228.82, with an average of 148.67, and spheroid sizes ranged between 509.84 and 36,118.42 square micrometers, with an average of 5,382.2 square micrometers. Spheroid size for this population of spheroids in HSV-1-inoculated and acyclovir-treated cultures was significantly increased relative to mock-infected cultures (*P* < 0.05). Enlarged spheroids in HSV-1-inoculated and acyclovir-treated cultures (larger than the largest, 3,014.81-square-micrometer spheroid in mock controls) also included both HSV-1 protein-expressing spheroids (immunostaining intensity above the highest value of 183.66 detected in mock controls) and spheroids with no significant HSV-1 protein expression demonstrated by immunostaining intensity in the range of uninfected controls (Fig. 6C). Specifically, 25 of the 118 enlarged spheroids (21.1%) expressed HSV-1 proteins.

These findings indicate that HSV-1 protein expression is detectable in some but not all enlarged melanoma spheroids in HSV-1-infected 3D melanoma cultures.

## DISCUSSION

Three-dimensional tumor cell cultures provide a useful *in vitro* experimental platform to study many aspects of tumor growth and cancer therapy (14–22). HSV-1-mediated oncolytic therapy is an emerging cancer treatment modality with potential effectiveness against a variety of malignancies (1, 2, 7). To better understand the interaction of HSV-1 with neoplastic cells, in the current study we inoculated 3D cultures of human uveal melanoma cells with HSV-1. We have shown here that HSV-1 infection of 3D melanoma cultures leads to the destruction of many tumor cells but surprisingly also is associated with an enhanced growth of a subpopulation of invasive tumor cells.

Three-dimensional melanoma cultures were established by placing tumor cells on the surface of a Matrigel matrix, which was followed by the growth of tumor cells on the matrix surface and invasion of the Matrigel matrix by some tumor cells to form multicellular tumor spheroids within the matrix. When established 3D melanoma cultures were inoculated with HSV-1 by placing virus on the surface of cultures, virus reached and infected tumor cells present on the Matrigel surface as well as tumor cells that formed spheroids inside the matrix. Infection caused extensive death of melanoma cells growing on the surface of the 3D matrix and significantly decreased the number of tumor cell spheroids within the matrix. However, HSV-1 infection did not lead to a complete destruction of tumor cells in the 3D cultures during a 17-day observation period and HSV-1 infection promoted the growth of some melanoma cells within the matrix as determined by a significantly increased size of residual viable multicellular tumor spheroids in virus-inoculated 3D cultures at 17 days after virus inoculation. Acyclovir treatment inhibited HSV-1-induced tumor cell killing but did not block virus infection-induced increase in spheroid size. Although our observations were made in a 3D *in vitro* experimental system, our findings suggest that HSV-1-tumor cell interactions during oncolytic virotherapy *in vivo* may also be associated with the unintended promotion of the growth of some tumor cells. Furthermore, our findings raise the possibility that HSV-1 infection of neoplastic cells during natural infections or vaccinations may promote the growth of some tumor cells.

Our studies do not identify the specific mechanism(s) by which HSV-1 infection enhances the growth of some tumor cells in 3D cultures. Previous studies in our laboratory indicated that melanoma and other tumor cells grown in 3D are more resistant to HSV-1 infection than cells grown in 2D (monolayer) cultures and that HSV-1 may establish a quiescent infection in some melanoma cells in 3D cultures (12, 13, 23). Possible causes of increased virus resistance of 3D tumor cultures against HSV-1 include impaired spread of virus within the extracellular matrix (ECM), extracellular matrix-induced decrease in the expression of HSV-1 entry receptors, and decreased permissiveness of tumor cells to virus replication mediated by extracellular matrix components (12, 13, 23). Our current study confirmed the long-term resistance of 3D melanoma cultures against complete HSV-1-mediated destruction. Possible—and not mutually exclusive—mechanisms by which HSV-1 infection in 3D melanoma cultures could lead to increased tumor growth include both direct and indirect viral effects. It is possible that HSV-1 establishes a nonproductive, persistent, noncytolytic infection in some tumor cells within the matrix and this persistent infection induces increased growth of infected cells. In addition, it is also possible that HSV-1 cannot infect some melanoma cells within the 3D matrix (due to limitation of viral spread by the matrix or lack of viral entry receptors on some cells) but indirectly induces increased growth of these cells through viral or cellular mediators released from infected bystander cells. Our studies indicated that in HSV-1-inoculated 3D melanoma cultures, many melanoma spheroids remained negative for HSV-1 protein expression, consistent with both of the above possibilities. Our studies using acyclovir suggested that viral replication may not be required for virus infection-associated tumor growth enhancement.

It is possible that persistent, nonproductive, noncytolytic HSV-1 infection in some tumor cells could be associated with the expression of HSV-1 products that can induce increased tumor cell growth. Naturally occurring latent HSV-1 infections of neurons demonstrate that long-term noncytolytic interaction of HSV-1 with cells is possible (reviewed in references 26, 27, and 28). HSV-1 latent infection in neurons is associated with the expression of some viral RNAs but not with an abundant expression of HSV-1 proteins (reviewed in references^26^, ^27^, and 28). Interestingly, latency-associated transcripts (LATs) expressed during latency have been shown to have a variety of effects on cells, including the inhibition of apoptosis (reviewed in references 26, 27, and 28). If noncytolytic persistent HSV-1 infection of melanoma cells occurs in 3D melanoma cultures, it is possible that LATs are expressed and exert an antiapoptotic effect on tumor cells leading to increased-sized spheroids. In addition to viral RNAs, the marginal expression of a variety of HSV-1 proteins with known capacity to induce cellular transcription factors promoting growth (26, 29–32) during noncytolytic persistent HSV-1 infection of melanoma cells could also play a role in infection-induced increased spheroid size. Interestingly, UV-irradiated HSV-1 has been shown to be capable of oncogenic transformation of nonneoplastic cells in 2D cultures (33). Transformation could not be observed with wild-type virus, as it destroyed the cultured cells through lytic infection, also suggesting that noncytolytic HSV infections could have a capacity of oncomodulation.

Increased growth of some melanoma cells in our 3D cultures could also be due to cellular or viral mediators released from infected cells that could induce increased growth of uninfected bystander cells. For instance, HSV-1 has been reported to induce cellular DNA synthesis in uninfected cells when uninfected and infected Vero cells are separated by a filter, in a dual-chamber setup, excluding HSV-1 transfer (34). *In vivo* studies have also shown that HSV-1 can induce VEGF-A production in infected corneal epithelial cells causing increased vascular growth (35).

It is clear that further studies are necessary to define the actual mechanisms by which HSV-1 infection induces increased growth in a subpopulation of melanoma cells in 3D cultures. Extension of these studies to other tumor types, viral strains, and doses will be important. A better understanding of HSV-1-mediated oncomodulation in this 3D *in vitro* model could provide highly significant new information about the possible dangers of HSV oncolytic therapy *in vivo* and about fundamental mechanisms of viral oncomodulation during oncolytic virotherapy, natural HSV-1 infections, and HSV-1 vaccinations.

## MATERIALS AND METHODS

### Viruses.

Recombinant HSV-1 strain K26GFP (25) was amplified and quantitated as described elsewhere (12, 13, 23, 36). Cells infected with HSV-1 strain K26GFP exhibit punctate nuclear fluorescence at early times in the replication cycle, and at later times during infection, a generalized cytoplasmic and nuclear fluorescence, including fluorescence at the cell membranes, can be observed (25). K26GFP was shown to grow with wild-type virus characteristics in cell culture (25).

### Cells.

Uveal melanoma cells (OCM1) were maintained in Eagle's minimal essential medium (product number 11095-080; Gibco) supplemented with heat-inactivated 15% fetal bovine serum (Fisher, Ontario, Canada) without the addition of exogenous extracellular matrix (ECM) molecules or growth factors. These cell lines were described in detail earlier (21, 37).

### Establishment of 3D uveal melanoma cultures and virus inoculation.

To establish 3D uveal melanoma cultures, ECM rich in laminin (Matrigel; BD Biosciences, Bedford, MA) was poured onto 12-well tissue culture plates to a depth of approximately 0.2 mm, followed by polymerization for 1 h at 37°C. Melanoma cells in Eagle's minimal essential medium (product number 11095-080; Gibco) were placed on the surface of Matrigel matrix and were incubated at 37°C for 4 days, with refreshing of culture medium every second day, and observed under an inverted microscope (Leica, Bannockburn, IL) daily. After 4 days of plating, melanoma cells grew on the Matrigel surface and invaded the matrix to form multicellular spheroids.

### HSV-1 inoculation of establishment of 3D uveal melanoma cultures and observation of virus spread in cultures.

After 4 days of plating, melanoma cells grew on the Matrigel surface and invaded the matrix to form multicellular spheroids. Culture medium was then removed, and the established 3D cultures were virus inoculated with HSV-1 (K26GFP) by dropping virus suspended in phosphate-buffered saline (PBS) at a calculated multiplicity of infection (MOI) of 0.5 PFU/cell on the surface of cultures and incubated for 1 h at 37°C. Control cultures were mock infected with 0.5 ml of sterile PBS and incubated for 1 h at 37°C. Following the viral inoculation or mock infection, the cultures were incubated and maintained with culture medium, with refreshing of the medium every 2 days. Some viruses and mock-infected 3D cultures were exposed to and maintained in 100 μM acyclovir (an antiviral agent that inhibits HSV-1 DNA replication) following the virus or mock-inoculation procedures (38, 39). All cultures were observed daily using an inverted fluorescence microscope for cell growth and GFP expression. The percentages of tumor cells expressing GFP at selected days after virus or mock infection were determined by manual counts in at least 10 representative high-power microscopic fields in at least two parallel cultures for each treatment type, and average expression per treatment type was established. GFP expression and tumor culture morphology were also documented by photography. Cell death in cultures was monitored by uptake of the charged cationic dye Trypan blue after incubation of cultures with Trypan blue (0.2%) for 10 min at 37°C.

### Fixation of 3D melanoma cultures and preparation of sections of formalin-fixed paraffin-embedded tumor cultures for morphological and immunohistochemical analysis.

Seventeen days after virus or mock infection, 3D cultures were fixed in 2% paraformaldehyde overnight at room temperature. After fixation, 3D cultures were removed from the plates and were maintained in 70% ethanol until paraffin embedding and sectioning. Sections (5 μm) of 3D cultures were deparaffinized and either stained with hematoxylin & eosin (H&E) for histologic examination and morphometric studies or processed for immunohistochemistry for HSV-1 protein expression.

### Analysis of multicellular tumor spheroid size and number.

For morphometric studies, H&E-stained sections of 3D melanoma cultures were scanned in using an Aperio AT2 scanner. The sizes of multicellular tumor spheroids formed by melanoma cells in cultures were determined by the analysis of photographic images captured at a magnification of ×20 in ImageScope. We determined a standard pixel count in ImageJ, for a specific area, in ImageScope at a magnification of ×20, finding that 10,000 pixels in ImageJ equaled 2,514.0196 square micrometers. The standard area pixel count was achieved by drawing a 50.14-μm by 50.14-μm square in ImageScope and then analyzing the area enclosed by the square in ImageJ, resulting in an enclosed-area pixel count. The area of melanoma spheroids was calculated by measuring the number of pixels within each spheroid and comparing it to the known standard as detailed above. Average spheroid size was determined for all treatment groups. The average number of spheroids per culture was calculated by determining the average number of spheroids per 500-μm by 500-μm area of Matrigel matrix in scanned images. In brief, scanned images of 3D melanoma cultures collected after various treatments were analyzed by counting cell aggregates containing more than two cells per designated 500-μm by 500-μm areas with complete analysis of the matrix areas of the cultures and calculating the average spheroid number per area for each treatment type.

The average number of nuclei (nuclear profiles) per spheroid in HSV-1 infected, HSV-1-infected and acyclovir-treated, and mock-infected 3D cultures 17 days following HSV-1 or mock infection was determined using scanned images of H&E-stained sections of 3D melanoma cultures after the various treatments. Nuclei were identified in images by morphology, and the number of nuclei was determined in at least 75 randomly selected spheroids per treatment group. The calculated average numbers of nuclei per spheroid were compared among treatment groups and analyzed by Welch's analysis of variance (ANOVA) along with a Games-Howell significance test.

### Analysis of HSV protein expression in spheroids.

To determine if HSV-1 proteins were expressed in 3D melanoma cultures following HSV-1 inoculation, 5-μm sections of 3D melanoma cultures were studied for HSV-1 protein expression by HSV-1 protein immunohistochemistry using a polyclonal anti-HSV-1 antibody (Dako) as described previously (40). HSV-1 protein expression was visualized as brown staining under a light microscope, photographed using Image Scope and analyzed in Image J to determine the strength of immunostaining. Immunostaining intensity for spheroid profiles was determined for whole spheroid profiles and not for the individual cells making up spheroids.

### Analysis of HSV protein expression by spheroid size.

HSV-1 protein expression in spheroids of virus-inoculated 3D cultures as a function of spheroid size was calculated using data generated for strength of HSV-1 protein expression for each spheroid and spheroid size. Data were assembled into scatter plots and were analyzed statistically by Welch's ANOVA along with a Games-Howell significance test.

## References

[1] Fukuhara H, Ino Y, Todo T 2016 Oncolytic virus therapy: a new era of cancer treatment at dawn. Cancer Sci 107:1373–1379. doi:10.1111/cas.13027. 27486853 PMC5084676

[2] Kuruppu D, Tanabe KK 2015 HSV-1 as a novel therapy for breast cancer meningeal metastases. Cancer Gene Ther 22:506–508. doi:10.1038/cgt.2015.43. 26384139 PMC4766812

[3] Chiocca EA 2008 The host response to cancer virotherapy. Curr Opin Mol Ther 10:38–45. 18228180

[4] Fukuhara H, Todo T 2007 Oncolytic herpes simplex virus type 1 and host immune responses. Curr Cancer Drug Targets 7:149–155. doi:10.2174/156800907780058907. 17346106

[5] Miller CG, Fraser NW 2003 Requirement of an integrated immune response for successful neuroattenuated HSV-1 therapy in an intracranial metastatic melanoma model. Mol Ther 7:741–747. doi:10.1016/S1525-0016(03)00120-5. 12788647 PMC2661757

[6] Toda M, Rabkin SD, Kojima H, Martuza RL 1999 Herpes simplex virus as an in situ cancer vaccine for the induction of specific anti-tumor immunity. Hum Gene Ther 10:385–393. doi:10.1089/10430349950018832. 10048391

[7] Andtbacka RHI, Kaufman HL, Collichio F, Amatruda T, Senzer N, Chesney J, Delman KA, Spitler LE, Puzanov I, Agarwala SS, Milhem M, Cranmer L, Curti B, Lewis K, Ross M, Guthrie T, Linette GP, Daniels GA, Harrington K, Middleton MR, Miller WH Jr, Zager JS, Ye Y, Yao B, Li A, Doleman S, VanderWalde A, Gansert J, Coffin RS 2015 Talimogene laherparepvec improves durable response rate in patients with advanced melanoma. J Clin Oncol 25:2780–2788. doi:10.1200/JCO.2014.58.3377. 26014293

[8] Kolodkin-Gal D, Zamir G, Edden Y, Pikarsky E, Pikarsky A, Haim H, Haviv YS, Panet A 2008 Herpes simplex virus type 1 preferentially targets human colon carcinoma: role of extracellular matrix. J Virol 82:999–1010. doi:10.1128/JVI.01769-07. 17977977 PMC2224594

[9] McKee TD, Grandi P, Mok W, Alexandrakis G, Insin N, Zimmer JP, Bawendi MG, Boucher Y, Breakfield XO 2006 Degradation of fibrillar collagen in a human melanoma xenograft improves the efficacy of an oncolytic herpes simplex virus vector. Cancer Res 66:2509–2513. doi:10.1158/0008-5472.CAN-05-2242. 16510565

[10] Nagano S, Perentes JY, Jain RK, Boucher Y 2008 Cancer cell death enhances the penetration and efficiency of oncolytic herpes simplex virus in tumors. Cancer Res 68:3795–3802. doi:10.1158/0008-5472.CAN-07-6193. 18483263 PMC2871708

[11] Vaha-Koskela M, Hinkkanen A 2014 Tumor restrictions to oncolytic virus. Biomedicines 2:163–194. doi:10.3390/biomedicines2020163. 28548066 PMC5423468

[12] Valyi-Nagy K, Dosa S, Kovacs SK, Bacsa S, Voros A, Shukla D, Folberg R, Valyi-Nagy T 2010 Identification of virus resistant tumor cell subpopulations in three-dimensional uveal melanoma cultures. Cancer Gene Ther 17:223–234. doi:10.1038/cgt.2009.73. 19893596

[13] Valyi-Nagy K, Folberg R, Valyi-Nagy T, Maniotis AJ 2007 Susceptibility of uveal melanoma to herpes simplex virus type 1: the role of tumor invasiveness, the extracellular matrix and chromatin sequestration. Exp Eye Res, 84:991–1000. doi:10.1016/j.exer.2007.01.023. 17386925 PMC1950675

[14] Abbott A 2003 Biology's new dimension. Nature 424:870–872. doi:10.1038/424870a. 12931155

[15] Friedrich MJ 2003 Studying cancer in 3 dimensions. JAMA 290:1977–1979. doi:10.1001/jama.290.15.1977. 14559935

[16] Ghosh S, Spagnoli GC, Martin I, Ploegert S, Demougin P, Heberer M, Reschner A 2005 Three-dimensional culture of melanoma cells profoundly affects gene expression profile: a high density oligonucleotide array study. J Cell Physiol 204:522–531. doi:10.1002/jcp.20320. 15744745

[17] Schmeichel KL, Bissell MJ 2003 Modeling tissue-specific signaling and organ function in three dimensions. J Cell Sci 116:2377–2388. doi:10.1242/jcs.00503. 12766184 PMC2933213

[18] Smalley KS, Lioni M, Herlyn M 2006 Life isn't flat: taking cancer biology to the next dimension. In Vitro Cell Dev Biol Anim 42:242–247. doi:10.1290/0604027.1. 17163781

[19] Nelson CM, Bissell MJ 2005 Modeling dynamic reciprocity: engineering three-dimensional culture models of breast architecture, function, and neoplastic transformation. Semin Cancer Biol 15:342–352. doi:10.1016/j.semcancer.2005.05.001. 15963732 PMC2933210

[20] Xu F, Burg KJL 2007 Three-dimensional polymeric system for cancer cell studies. Cytotechnology 54:135–143. doi:10.1007/s10616-007-9065-4. 19003005 PMC2267509

[21] Maniotis AJ, Valyi-Nagy K, Karavitis J, Moses J, Boddipali JV, Wang Y, Nuñez R, Setty S, Arbieva Z, Bissell MJ, Folberg R 2005 Chromatin organization measured by AluI restriction enzyme changes with malignancy and is regulated by the extracellular matrix and the cytoskeleton. Am J Pathol 166:1187–1203. doi:10.1016/S0002-9440(10)62338-3. 15793298 PMC1602386

[22] Folberg R, Arbieva Z, Moses J, Hayee A, Sandal T, Kadkol S, Lin AY, Valyi-Nagy K, Setty S, Leach L, Chévez-Barrios P, Larsen P, Majumdar D, Pe'er J, Maniotis AJ 2006 Tumor cell plasticity in uveal melanoma–micro-environment directed dampening of the invasive and metastatic genotype and phenotype accompanies the generation of vasculogenic mimicry patterns. Am J Pathol 169:1376–1389. doi:10.2353/ajpath.2006.060223. 17003493 PMC1698855

[23] Voros A, Kormos B, Valyi-Nagy T, Valyi-Nagy K 2013 Increased resistance of breast, prostate and embryonal carcinoma cells against herpes simplex virus in three dimensional cultures. ISRN Oncol 2013: 104913. doi:10.1155/2013/104913. 24455304 PMC3885282

[24] Zhu Y, Yang Y, Guo J, Dai Y, Ye L, Qiu J, Zeng Z, Wu X, Xing Y, Long Y, Wu X, Ye L, Wang S, Li H 2017 Ex vivo 2D and 3D HSV-2 infection model using human normal vaginal epithelial cells. Oncotarget 8:15267–15282. doi:10.18632/oncotarget.14840. 28146426 PMC5362485

[25] Desai P, Pearson S 1998 Incorporation of the green fluorescent protein into the herpes simplex virus type 1 capsid. J Virol 72:7563–7568. 9696854 10.1128/jvi.72.9.7563-7568.1998 PMC110002

[26] Roizman B, Zhou G, Du T 2011 Checkpoints in productive and latent infections with herpes simplex virus 1: conceptualization of the issues. J Virol 85:7474–7482. doi:10.1128/JVI.00180-11. 22052379

[27] Valyi-Nagy T, Shukla D, Engelhard HH, Kavouras J, Scanlan P 2007 Latency strategies of alphaherpesviruses: herpes simplex virus and varicella-zoster virus latency in neurons, p 1–36. *In* Minarovits J, Gonczol E, Valyi-Nagy T (ed), Latency strategies of herpesviruses. Springer, New York, NY.

[28] Knipe DM 2015 Nuclear sensing of viral DNA, epigenetic regulation of herpes simplex virus infection, and innate immunity. Virology 479-480: 153–159. doi:10.1016/j.virol.2015.02.009. 25742715 PMC4424148

[29] Amici C, Belardo G, Rossi A, Santoro MG 2001 Activation of I kappa b kinase by herpes simplex virus type 1: a novel target for anti-herpetic therapy. J Biol Chem 276:28759–28766. 11387335 10.1074/jbc.M103408200

[30] McLean TI, Bachenheimer SL 1999 Activation of cJUN N-terminal kinase by herpes simplex virus type 1 enhances viral replication. J Virol 73:8415–8426. 10482593 10.1128/jvi.73.10.8415-8426.1999 PMC112860

[31] Valyi-Nagy T, Deshmane SL, Dillner AJ, Fraser NW 1991 Induction of cellular transcription factors in trigeminal ganglia of mice by corneal scarification, HSV-1 infection and explantation of trigeminal ganglia. J Virol 65:4142–4152. 1649322 10.1128/jvi.65.8.4142-4152.1991 PMC248848

[32] Zachos G, Clements B, Conner J 1999 Herpes simplex virus type 1 infection stimulates p38/c-Jun N-terminal mitogen-activated protein kinase pathways and activates transcription factor AP-1. J Biol Chem 274:5097–5103. doi:10.1074/jbc.274.8.5097. 9988758

[33] Duff R, Rapp F 1973 Oncogenic transformation of hamster embryo cells after exposure to inactivated herpes simplex virus type 1. J Virol 12:209–217. 4355928 10.1128/jvi.12.2.209-217.1973 PMC356614

[34] Schmidt N, Hennig T, Serwa RA, Marchetti M, O'Hare P 2015 Remote activation of host cell DNA synthesis in uninfected cells signaled by infected cells in advance of virus transmission. J Virol 89:11107–11115. doi:10.1128/JVI.01950-15. 26311877 PMC4621119

[35] Wuest TR, Carr DJJ 2010 VEGF-A expression by HSV-1–infected cells drives corneal lymphangiogenesis. J Exp Med 207:101–115. doi:10.1084/jem.20091385. 20026662 PMC2812544

[36] Akhtar J, Tiwari V, Oh M, Kovacs M, Jani A, Kovacs SK, Valyi-Nagy T, Shukla D 2008 HVEM and nectin-1 are the major mediators of herpes simplex virus 1 (HSV-1) entry into human conjunctival epithelium. Invest Ophthalmol Vis Sci 49:4026–4035. doi:10.1167/iovs.08-1807. 18502984 PMC2569872

[37] Folberg R, Kadkol S, Frenkel S, Valyi-Nagy K, Jager MJ, Pe'er J, Maniotis AJ 2008 Authenticating cell lines in ophthalmic research laboratories. Invest Ophthalmol Vis Sci 49:4697–4701. doi:10.1167/iovs.08-2324. 18689700 PMC2576485

[38] Thellman NM, Botting C, Madej Z, Triezenberg SJ 2017 An immortalized human dorsal root ganglion cell line provides a novel context to study herpes simplex virus 1 latency and reactivation. J Virol 91:e00080-17. doi:10.1128/JVI.00080-17. 28404842 PMC5446634

[39] Kobayashi M, Kim JY, Camarena V, Roehm PC, Chao MV, Wilson AC, Mohr I 2012 A primary neuron culture for the study of herpes simplex virus latency and reactivation. J Vis Exp 2012:3823. doi:10.3791/3823. PMC3466666 22491318

[40] Kavouras JH, Prandovszky E, Valyi-Nagy K, Kovacs SK, Tiwari V, Kovacs M, Shukla D, Valyi-Nagy T 2007 HSV-1 infection induces oxidative stress and the release of bioactive lipid peroxidation by-products in P19N mouse neural cell cultures. J Neurovirol 13:416–425. doi:10.1080/13550280701460573. 17994426

